# The Symbiotic Effect of a New Nutraceutical with Yeast β-Glucan, Prebiotics, Minerals, and *Silybum marianum* (Silymarin) for Recovering Metabolic Homeostasis via *Pgc-1α*, *Il-6*, and *Il-10* Gene Expression in a Type-2 Diabetes Obesity Model

**DOI:** 10.3390/antiox11030447

**Published:** 2022-02-23

**Authors:** Aline Boveto Santamarina, Ruan Carlos Macêdo Moraes, Victor Nehmi Filho, Gilson Masahiro Murata, Jéssica Alves de Freitas, Danielle Araujo de Miranda, Anderson Romério Azevedo Cerqueira, Soraia Katia Pereira Costa, Ana Flávia Fernandes Ferreira, Luiz Roberto Britto, Juliana Alves de Camargo, Daniela Rodrigues de Oliveira, Flavia Neto de Jesus, José Pinhata Otoch, Ana Flávia Marçal Pessoa

**Affiliations:** 1 Department of Biosciences, Federal University of São Paulo (UNIFESP), Santos 11015-020, SP, Brazil; alinesantamarina@gmail.com; 2 Natural Products and Derivatives Laboratory (LIM-26), Department of Surgery, University of São Paulo Medical School, São Paulo 01246-903, SP, Brazil; moraes.ruan@usp.br (R.C.M.M.); victor@nehmi.com.br (V.N.F.); jessica.freitas@fm.usp.br (J.A.d.F.); danielaoliveira.phd@gmail.com (D.R.d.O.); pinhata@usp.br (J.P.O.); 3 Research and Development Efeom Nutrition S/A, São Paulo 03317-000, SP, Brazil; 4 Laboratory of Medical Investigation (LIM-29), Clinic Medical Department, University of São Paulo Medical School, São Paulo 01246-903, SP, Brazil; gilson.murata@usp.br; 5 Department of Physiology, Escola Paulista de Medicina/Universidade Federal de São Paulo, São Paulo 04023-062, SP, Brazil; daniellea.miranda@gmail.com; 6 Departamento de Farmacologia, Instituto de Ciências Biomédicas, Universidade de São Paulo, São Paulo 05508-000, SP, Brazil; anderson.romerio@usp.br (A.R.A.C.); skcosta@usp.br (S.K.P.C.); 7 Departamento de Fisiologia, Instituto de Ciências Biomédicas, Universidade de São Paulo, São Paulo 05508-000, SP, Brazil; anaffernandesf@gmail.com (A.F.F.F.); britto@icb.usp.br (L.R.B.); 8 Laboratory of Medical Investigation (LIM-55), Urology Department, University of São Paulo Medical School, São Paulo 01246-903, SP, Brazil; ju.alvcam@gmail.com; 9 Genomic Sciences and Precision Medicine Center (GSPMC), Department of Surgery, Medical College of Wisconsin, Milwaukee, WI 53226, USA; 10 Department of Physiology and Pharmacology, Snyder Institute for Chronic Diseases, Cumming School of Medicine Alberta, Calgary, AB T2N 1N4, Canada; flavia.netodejesus@ucalgary.ca; 11 Brazilian Academic Consortium for Integrative Health (CABSIN), Natural Products Committee, São Paulo 05449-070, SP, Brazil

**Keywords:** nutraceutical, silymarin, prebiotic, yeast β-glucan, minerals, obesity, type-2 diabetes, *Interleukin-6*, *Pgc-1α*, antioxidant enzymes

## Abstract

The use of natural products and derivatives for the prevention and control of non-communicable chronic diseases, such as type-2 diabetes (T2D), obesity, and hepatic steatosis is a way to achieve homeostasis through different metabolic pathways. Thus, male C57BL/6 mice were divided into the following groups: high-fat diet (HFD) vehicle, HFD + Supplemented, HFD + Supplemented_S, and isolated compounds. The vehicle and experimental formulations were administered orally by gavage once a day over the four weeks of the diet (28 consecutive days). We evaluated the energy homeostasis, cytokines, and mitochondrial gene expression in these groups of mice. After four weeks of supplementation, only the new nutraceutical group (HFD + Supplemented) experienced reduced fasting glycemia, insulin, HOMA index, HOMA-β, dyslipidemia, ectopic fat deposition, and hepatic fibrosis levels. Additionally, the PPARγ coactivator 1 α (*Pgc-1α*), interleukin-6 *(Il-6),* and interleukin-10 *(Il-10*) gene expression were augmented, while hepatic steatosis decreased and liver parenchyma was recovered. The glutathione-S-transferase activity status was found to be modulated by the supplement. We discovered that the new nutraceutical was able to improve insulin resistance and hepatic steatosis mainly by regulating *IL-6, IL-10*, and *Pgc-1α* gene expression.

## 1. Introduction

Nowadays, the search for non-pharmacological health-promoting supplements is a trend. Pursuit of natural compounds applied in chronic non-communicable diseases’ treatment and prevention has been growing as a research field in recent decades, aiming to promote not only a long lifespan but also better quality of life over time. In this sense, the use of conventional medications has given way to the growth of natural integrative therapies with few side effects, using food supplements and nutraceuticals [1,2]. Indeed, recent research has shown that non-pharmacological interventions can be used as strategies for the prevention and treatment of inflammatory and metabolic diseases, such as obesity, metabolic syndrome, dyslipidemia, and type-2 diabetes (T2D) [3,4,5].

At the same time, the incidence of obesity has grown over the last decades. Its multifactorial etiology involves genetic and environmental factors, such as an unhealthy diet and sedentary lifestyle [6]. It is well-established that the consumption of a high-fat diet contributes to the development of several non-communicable chronic diseases, including T2D and metabolic syndrome [7,8].

In obesity, the classic chronic low-grade inflammatory state, also known as meta inflammation, is responsible for the recruitment and activation of T lymphocytes and macrophages in adipose tissue. In turn, there is an increased occurrence of proinflammatory cytokine and reactive oxygen species (ROS). Hyperglycemia and hyperinsulinemia are known to trigger an inflammatory process and oxidative stress environment [9]. These cellular pathways activated by pro-inflammatory cytokines directly influence the insulin signaling in insulin target tissues like the liver and eventually lead to T2D [10]. In parallel, insulin resistance is also established by visceral fat deposition, which increases the activation of the lipolysis process [11,12]. In turn, portal vein free fatty acids’ (FFAs’) flow increases, exposing the liver to a greater influx of FFAs modifying hepatic metabolic patterns, contributing to the maintenance of systemic hyperinsulinemia and insulin resistance [13]. On the other hand, hepatic fatty acid oxidation in obesity is reduced by mitochondrial dysfunctions triggered by obesity, resulting in hepatic accumulation of triacylglycerols [14,15]. Thus, non-alcoholic fatty liver disease (NAFLD) has become the most common cause of chronic liver disease, leading to non-alcoholic steatohepatitis (NASH) [16]. NAFLD is closely related to an increase in metabolic syndrome and T2D in the obese population [17,18]. Thus, NASH is frequently considered the hepatic outcome of type-2 diabetes. Indeed, insulin resistance also plays a central role in the development of a fatty liver [19].

A well-recognized mechanism of obesity-related insulin resistance is associated with the functional deficiency of IRS-1 phosphorylation, which results in abnormal insulin action. In this sense, the suppressor of cytokine signaling (SOCS) seems to be involved in inhibiting insulin action through IRS-1, linking metabolic and immune system functioning under an insulin-resistance pathophysiology [20]. *Pgc-1α* (PPARγ coactivator 1 α) is known to act on mitochondrial biogenesis and reactive oxygen species (ROS); however, it also plays a modulatory role in cytokine expression, improving insulin resistance and hepatic steatosis [21]. Indeed, a hallmark of insulin resistance is unbalanced cytokine production. In this scenario, usually, interleukin-6 (*IL-6*) stands as a pro-inflammatory marker, alongside other classic cytokines like tumor necrosis factor-α (TNF-α) and interleukin 1β (IL-1β). Currently, the literature discusses how *IL-6* plays an ambivalent role, depending on the tissue and physiological situation. Its positive role is already known in muscle recovery after exercise and central nervous tissues such as the hypothalamus, for example. Therefore, the literature suggests a constitutive role of *IL-6*, promoting anti-inflammatory action via its pleiotropic cell type-specific effect in obesity meta-inflammation and its associated metabolic disorders like T2D [22].

Indeed, *IL-6* is a complex pleiotropic cytokine, with an in-depth tissue-dependent mechanistic function. Nonetheless, *IL-6* is still commonly related to T2D development since acute *IL-6* production seems to impair insulin action in mice [23]. Furthermore, recent studies demonstrate that pharmacological intervention to block *IL-6* secretion can exert beneficial effects on glucose homeostasis [10,24]. This reinforces the multilayered role of this cytokine and the need for further investigation into its T2D physiological role.

Moreover, the increased incidence and prevalence of insulin resistance and obesity-related T2D is a crisis that has been reported for decades by worldwide public-health regulators. Despite efforts to mitigate this syndrome, there has been little effect on the increase of any diseases associated with it. Research has been looking for modern solutions to solve a problem that mostly affects low-income populations [25]. In this context, it is also important to focus efforts on options that involve natural products at an affordable cost, to reach the population most affected by these chronic non-communicable diseases.

A recently explored way to modulate different metabolic pathways is through the intestinal microbiota. The importance of gut resident microorganisms and host health interaction is still largely unknown, and in this field, there is still a lot to be explored. However, it is already known that interventions with prebiotic molecules can reshape the intestinal microbiota with positive outcomes for metabolic diseases [26]. Therefore, strategies of microbiota reshaping with prebiotics, aiming to modulate insulin resistance and inflammation, might make for promising interventions [19]. The effect of isolated prebiotic compounds, such as fructo-oligosaccharides (FOS) [27], galacto-oligosaccharides (GOS) [28], and yeast β-glucans [29,30], has been shown to improve metabolic markers, thus recovering the health status in different models of metabolic diseases like T2D.

Likewise, protocols for mineral supplementation—including magnesium [31], zinc [32], and selenium [33]—as well as plant-derived compounds, like *Silybum marianum* (L.) Gaertn. (silymarin) [34], in isolation, have already demonstrated their benefits in the improvement of mitochondrial activity and key inflammatory molecules involved in the pathogenesis of several non-communicable chronic diseases such as T2D, obesity, and cardiovascular diseases. All these compounds together might have a symbiotic effect, working simultaneously with each other and favoring a potentiated effect when associated. Thus, they might represent a valuable tool for non-pharmacological interventions [5].

In this study, we propose the use of a supplement containing β-glucan, prebiotics, minerals, and *Silybum marianum* seed extract (silymarin) (milk thistle or silymarin) to modulate the inflammatory response and metabolism of obese mice or T2D mice [35]. We hypothesized that the symbiotic interaction among these products would improve the absorption of nutrients, triggering a decrease in fatty liver diseases through inflammatory pathways and oxidative stress modulation, thus improving insulin sensitivity.

## 2. Materials and Methods

### 2.1. Supplement Composition

The formulation of the supplement (patent number: BR 10 2020 016,156 3) developed and tested in the present study contained the following components: zinc (Zn) 0.63%; selenium (Se) 0.003%; magnesium (Mg) 4.35%; FOS 49.69%; GOS 31.05%, and 1.3/1.6-(β glycosidic bonds) yeast β-glucans (*Saccharomyces cerevisiae*) 11.18% (Yes Sinergy, Campinas, São Paulo, Brazil), along with silymarin (3.11%) extract from the *Silybum marianum* seed (Ningbo Vitax Biotech Co., Ningbo, China). The percentages of each of the minerals were calculated based on the dietary reference values for nutrients published by the European Food Safety Authority [36]. The percentages of silymarin extract [34], FOS [37] GOS [38], and yeast β-glucans [39] were based on previous studies and considered the body area of the animals, determined by the equation: human equivalent dose (mg/kg) = animal dose (mg/kg) 12.33 [40]. The final product was diluted in mineral water using 2% carboxymethyl cellulose as the emulsifier. The supplement composition offered to each group is described in Table 1.

### 2.2. Animal Model and Oral Supplementation

Sixty-day-old adult male C57BL/6 mice were obtained from the Central Vivarium of Mice at the University of São Paulo School of Medicine (FMUSP). All mice were maintained in a temperature-controlled room at 24 ± 2 °C and subjected to a 12 h light/12 h dark cycle. The animals were subsequently divided into two groups: control and obese. The control group was fed a standard nonfat diet (CTL) that contained 3.54 kcal/g. The other group (called obese) consumed a high-fat diet (HFD) that contained 5.25 kcal/g, which was composed of 30% saturated fat (mainly lard), 15.95% carbohydrate, and 20% protein [41] (Prag Soluções Biosciences, Jaú, São Paulo, Brazil). The diet composition is presented in Supplementary Table S1. The mice were placed on these diets for 14 weeks and were allowed to eat the chow *ad libitum*. During week 10, the mice were further divided into the following groups: HFD + Vehicle, the new nutraceutical—HFD + Supplemented, the new supplement without *Silybum marianum* (Silymarin extract)—HFD + Supplemented_S, HFD + *Silybum marianum* (Silymarin extract), HFD + Prebiotics, and HFD + Minerals (n = 5–9 per group).

**Figure 1:**
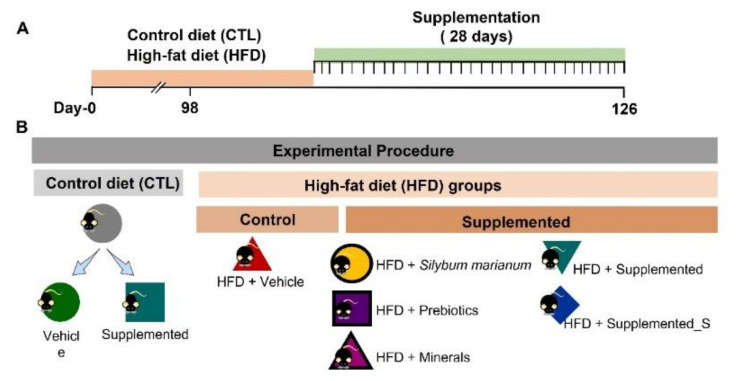
Schematic outline of the experimental procedure and supplementation time, common to all animals. (**A**) Timeline; (**B**) experimental groups.

The vehicle and experimental formulation were administered daily by gavage to ensure that all animals ingested the same amount of supplement for the four weeks (28 consecutive days) of the experimental protocol. We chose this treatment period to assess the long-term effect of supplementation on control and obese animals. The animals were euthanized four weeks after supplementation with an excessive dose of ketamine and xylazine. All experiments were conducted following the National Institutes of Health guidelines. The protocols were reviewed and approved by the USP-FMUSP Ethics Committee (numbers: 1185/2018 and 1519/2020) according to the protocol described in Figure 1.

### 2.3. Food Consumption and Body Parameters

The food intake, water, and feeding efficiency were estimated using the following formulas [42]: food intake = initial weight of food provided—final weight of food recovered (g); water intake = initial volume of water provided—final volume of water recovered (mL); feed conversion efficiency (FCE) = body weight gain (g)/mean food intake. These values were calculated for each cage and then divided by the number of animals in the cage (two or three animals per cage). Additionally, the body mass index was calculated using the following formula: body mass index = body weight (g)/naso-anal length (cm)^2^. The naso-anal length was used to normalize the weekly body weight gain and final body weight [43]. The food and water intake and animal weight were recorded twice a week during the supplementation.

### 2.4. Biochemistry Parameters

The levels of cholesterol, triglycerides, total proteins, albumin, and globulins in the plasma were measured with a commercially available kit (Bioclin, Belo Horizonte, MG, Brazil). The low-density lipoprotein cholesterol (LDL-c) and very-low-density lipoprotein cholesterol (VLDL-c) levels were calculated according to Friedewald et al. [44]. Blood samples were collected from the tail and glycemia was measured with an Accu-Chek Active blood glucose monitor (Roche, Mannheim, Germany) [42]. Insulin was determined using a rat/mouse insulin enzyme-linked immunosorbent assay (ELISA) kit from Millipore/Sigma-Aldrich (catalog number EZRMI13K-St Charles, MO, USA). The Homeostatic Model Assessment for Insulin Resistance (HOMA-IR) was calculated using the following formula: HOMA-IR (mmol/L) = fasting glucose value (mg/dL) × fasting insulin value (ng/dL)/405 [45]. The β-cell function was obtained by the HOMA-β method using the following formula: HOMA-β = 20 × fasting insulin value (µU/mL)/fasting glucose value (mmoL/L) −3.5) [46].

### 2.5. Antioxidant Enzyme Activity Assay

The liver samples were weighed, homogenized in phosphate buffer (50 mM; pH 7.0) in the proportion of 1:10, centrifuged (10,000 rpm, 4 °C, 10 min), and the supernatant was separated for enzymatic measurement.

The superoxide dismutase (SOD) activity was determined by the formation of the XTT-formazan product. The measured reaction occurs between xanthine, xanthine oxidase, and SOD, generating the superoxide radical anion (O_2_^•−^). This, in turn, reduces the XTT reagent (Sigma; St. Louis, MO, USA) to the XTT-formazan product, which absorbs light at 470 nm o-dianisidine (OD). SOD hijacks O_2_^•−^ and reduces the formation of the XTT-formazan product. The result was expressed as SOD units (USOD)/mg of protein. The SOD unit was defined as the amount of SOD capable of transforming 1 µmol/min of O_2_.

The GST (glutathione S-transferase) activity was based on the generation of a complex between GSH and 1-chloro-2,4-dinitrobenzene (CDNB; Sigma; St. Louis, MO, USA), catalyzed by GST^2^. The increase in absorbance was directly proportional to the GST activity in the sample, which was measured under o-dianisidine (OD) equal to 340 nm for 30 min at a temperature of 25 °C. The samples were analyzed in duplicate, and the results were expressed as µmol GSH conjugate/min/mg protein.

Glutathione reductase (GR) detection was based on a direct measure of GR activity, which used NADPH as a cofactor in the reduction of GSSG in GSH. The oxidative reaction from NADPH to NADP+ was measured via absorbance decay under o-dianisidine (OD) equal to 340 nm at 37 °C. The samples were analyzed in duplicate and expressed as µmol NADPH/min/mg protein. The catalase (CAT) activity was assessed after diluting the sample (1:100) in 50 mM phosphate buffer. The method involves two reactions: (1st) H_2_O_2_ (10 nM) undergoes dismutation by tissue catalase for 10 min at room temperature. This reaction is stopped by the addition of NaN3 (1 mM); (2nd) the remaining H_2_O_2_ is determined by oxidation of the o-dianisidine reagent (OD; 0.167 mg/mL; Sigma; St. Louis, MO, USA) in a reaction catalyzed by the enzyme peroxidase HRP (horseradish peroxidase; 0.095 mg/mL; Sigma; St. Louis, MO, USA) at pH 6.0. The speed of the o-dianisidine oxidation product was monitored by the increase in absorbance at 460 nm (Spectra Max Plus 384, Molecular Devices Inc.; Sunnyvale, CA, USA) for 10 min. To inactivate the catalase (reaction blank), supernatants were incubated at 60 °C for 2 h. The catalase activity value was calculated from the maximum speed per minute of each reaction and extrapolated on the H_2_O_2_ curve. The standard H_2_O_2_ curve (8820–11.3 µM) was performed and the results were expressed in catalase units (UCAT)/mg protein. A catalase unit was defined as the degradation of 1 µmol of H_2_O_2_ min^−1^ at 25 °C.

Glutathione peroxidase (GPx) activity was determined by indirect measurement of GPx activity, through a reaction associated with GR. Oxidized glutathione (GSSG), produced by reduction via hydroperoxides by GPx, was recycled to generate its reduced state by GR (Sigma; St. Louis, MO, USA) and NADPH (Sigma; St. Louis, MO, USA). The substrate used was tert-butyl hydroperoxide. The oxidation of NADPH to NADP+ was accompanied by a decrease in absorbance at 340 nm at 37 °C. The samples were analyzed in duplicate and expressed as µmol GSH/min/mg protein.

### 2.6. Oil Red O Staining

Oil red O was used to stain neutral triacylglycerols and lipids on the liver. Thirty-eight liver samples (n = 3–5/group) were immersed in a tissue freezing medium (Tissue-Tek OCT Compound—Sakura Finetek, Torrance, CA, USA). Sections of 10 µm were cut in a cryostat (Leica CM3050 S Research Cryostat). Liver right lobe tissue was immersed in 30% sucrose (Labsynth, Diadema, São Paulo, Brazil) in PBS overnight or until the tissue sank. Oil red O was prepared by diluting a stock solution (0.5 g oil red O—Sigma O0625—in 100 mL isopropanol) in distilled water (2:3 [vol/vol]), followed by filtration. The tissue was stained with oil red O for 20 min. After washing in distilled water, tissue was counterstained with hematoxylin for five minutes. Liver slides were then photographed with a microscope attached to a desktop (Leica Microsystems DMC2900, São Paulo, São Paulo, Brazil) through the LAS V4.6 program (Leica Microsystems). Quantifying tissue lipid accumulation was made according to [47].

### 2.7. Hematoxylin-Eosin (HE) Staining

Hematoxylin-eosin was used to stain nucleic acids in blue (hematoxylin) and the cytoplasm and extracellular matrix in pink (eosin) [48]. Thirty-eight livers (n = 3–5/group) were immersed in a tissue freezing medium (Tissue-Tek OCT Compound—Sakura Finetek USA). Sections of 10µm were cut in a cryostat (Leica CM3050 S Research Cryostat). The tissue was washed in distilled water for 10 min. Hematoxylin (EasyPath, EP-101071) was filtrated before use. The tissue was stained with hematoxylin for three minutes and washed for five minutes in distilled water. After the tissue was stained with eosin (EasyPath, EP-101061) for one minute, it was rapidly immersed into distilled water to remove excess eosin. The liver slides were then photographed with a microscope attached to a desktop (Leica Microsystems DMC2900, SP, Brazil) through the LAS V4.6 program (Leica Microsystems).

### 2.8. Real-Time qPCR

The liver samples were homogenized in TRIzol (Invitrogen, Carlsbad, CA, USA), and the total RNA was isolated following the manufacturer’s suggested protocol. The total RNA was reverse-transcribed using the Promega GoScript^TM^ Reverse Transcription System #A6010 (Madison, WI, USA) according to manufacturers’ instructions. qPCR was carried out with SYBR Green Real-Time Master Mix (Promega) following the manufacturer’s protocol using 10 ng of cDNA per sample, and 200 nM of each primer. Amplification and PCR product detection were performed with the ABI prism 7500 fast real-time PCR system (Applied Biosystems, CA, USA). The specificity of the SYBR^®^ green assay was confirmed by performing a melting-point analysis and comparing it with an *in-silico* curve designed in the program *uMelt^®^* (https://dna-utah.org/umelt/umelt.html, accessed on 19 May 2021). The primer sequences (http://www.ncbi.nlm.nih.gov/gene/ accessed on 13 April 2021) are described in Supplementary Table S2. The gene expression level normalized to the *B2m* and *Gapdh* [49] for liver samples was calculated using the ΔΔ*CT* method regarding CTL animals [50].

### 2.9. Statistics Analysis

The data were classified as parametric or nonparametric based on the Shapiro–Wilks test. Parametric data are expressed as mean ± standard deviation (SD) and nonparametric data as the median and interquartile range. For parametric data, comparisons between two groups were performed using the *t*-test with or without Welch’s correction. Comparisons of nonparametric data including two groups were performed employing the Mann–Whitney test. For comparisons > 2 groups, we performed analysis of variance (ANOVA) for data with a parametric distribution using Tukey’s post-hoc test. For nonparametric data, we used the Kruskal–Wallis test with Müller Dunn’s post-test. Means and standard deviations were used to generate the effect size estimates (i.e., Hedge’s g). The Hedge’s g effect-size estimate was generated given that it adjusts for the variation in sample sizes. Effect size estimates were adjusted for the sample size (Cohen’s d), and 95% confidence intervals were calculated to assess the statistical significance of average effect sizes [51]. Hedge’s g considers small, medium, and large effects (0.2, 0.5, and 0.8, respectively) [51]. Where there were significant differences between treatment groups, Hedges’ g statistic (the recommended measure for sample sizes < 20) was used to calculate the effect size of these differences [52].

Differences were considered statistically significant for α = 0.05. Statistical data analysis was carried out with the statistical package software GraphPad PRISM (GraphPad Prism version 5.0 for Windows, GraphPad Software, San Diego, CA, USA, www.graphpad.com, accessed on 2 November 2021).

All intervention experiments were conducted following the National Institutes of Health guidelines. The experimental protocols were submitted to the USP-FMUSP Ethics Committee and approved under the protocol numbers: 1185/2018 and 1519/2020.

## 3. Results

### 3.1. Effects of the New Nutraceutical Formulation on Body Measures and Plasmatic Biomarkers

**Figure 2:**
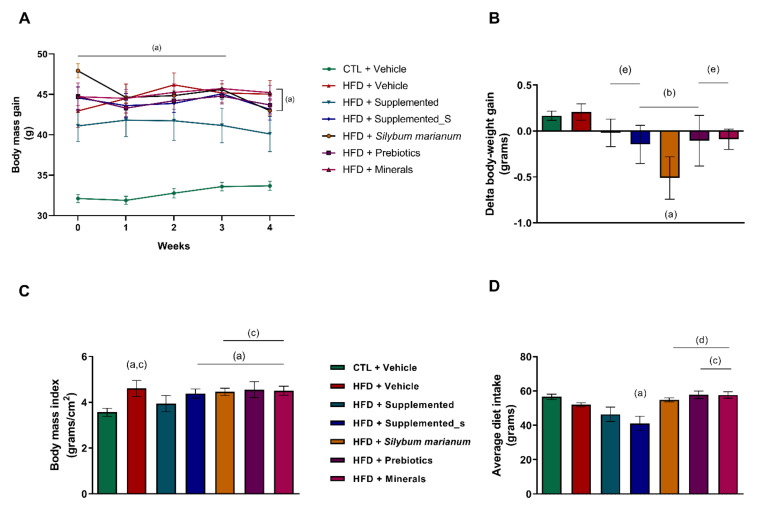
Body measurement data. (**A**) Weekly body mass gain; (**B**) Delta body weight gain; (**C**) Normalized body weight gain; (**D**) Average dietary intake. Significant *p* < 0.05 in one-way ANOVA followed by Tukey’s post-hoc test (n = 4–8 per group): (a) versus CTL + Vehicle; (b) versus HFD + Vehicle; (c) versus HFD + Supplemented; (d) versus HFD+Supplemented_S; (e) versus HFD+*Silybum marianum*. HFD = high-fat diet. Values are means ± SD.

The body mass was evaluated weekly. It was evident that the high-fat diet (HFD) protocol efficiently induced the experimental model of obesity since the control diet group (CTL + Vehicle) had significantly lower body mass gain in relation to all the HFD-fed groups (*p* < 0.0001 in all comparisons). However, it is noteworthy that the HFD + Supplemented group had significantly less weight gain from the third week onward compared to the other groups receiving the HFD diet (*p* = 0.0008 versus HFD + Vehicle; *p* = 0.0313 versus HFD + Supplemented_S; *p* < 0.0001 versus HFD + *Silybum marianum*; *p* = 0.0050 versus HFD + Prebiotics, and *p* < 0.0001 versus HFD + Minerals), as shown in Figure 2A.

Moreover, all the supplemented groups independently from the diet composition (CTL or HFD) showed a body weight loss during the experimental period, especially HFD + *Silybum marianum*, which had the greatest body mass reduction promoted by its supplementation compared to all the other experimental groups, despite the HFD challenge (*p* < 0.0001 versus CTL + Vehicle; *p* < 0.0001 versus HFD + Vehicle; *p* = 0.0003 versus HFD + Supplemented; *p* = 0.0208 versus HFD + Supplemented_S; *p* = 0.0016 versus HFD + Prebiotics, and *p* = 0.0026 versus HFD + Minerals), as displayed in Figure 2B. The body mass index (BMI) makes clear that only the HFD + Supplemented group had the BMI levels brought back to CTL diet levels. CTL + Vehicle had a lower BMI versus all HFD groups (*p* < 0.0001 in all comparisons) as well as the HFD + Supplemented group versus HFD + Vehicle (*p* = 0.0024), HFD + *Silybum marianum* (*p* = 0.0168), HFD + Prebiotics (*p* < 0.0028), and HFD + Minerals (*p* = 0.0139), while the HFD + Vehicle and the other HFD groups with different types of supplementation did not differ from each other (Figure 2C). Regarding the dietary intake, HFD + Supplemented_S had a lower dietary intake than the CTL + Vehicle group (p = 0.0028). The HFD + Supplemented group had a lower dietary intake compared both the HFD + Prebiotics (p = 0.0346) and HFD + Minerals (*p* = 0.0355) groups. The HFD + Supplemented_S group also displayed a lower dietary intake than the other HFD groups receiving interventions (HFD + *Silybum marianum p* = 0.0055, HFD + Prebiotics *p* = 0.0004, and HFD + Minerals *p* = 0.0005), as shown in Figure 2D.

Based on these results, it is possible to suggest that the supplementation contributed to the body mass reduction through increasing satiety, which was reflected in lower diet ingestion. Considering that the CTL diet demonstrated the well-established obesity scenario, from this point onwards, the data presented will consider only the HFD-fed groups since analyzing the effect of different supplementation in obesity was the main goal of this study.

**Figure 3:**
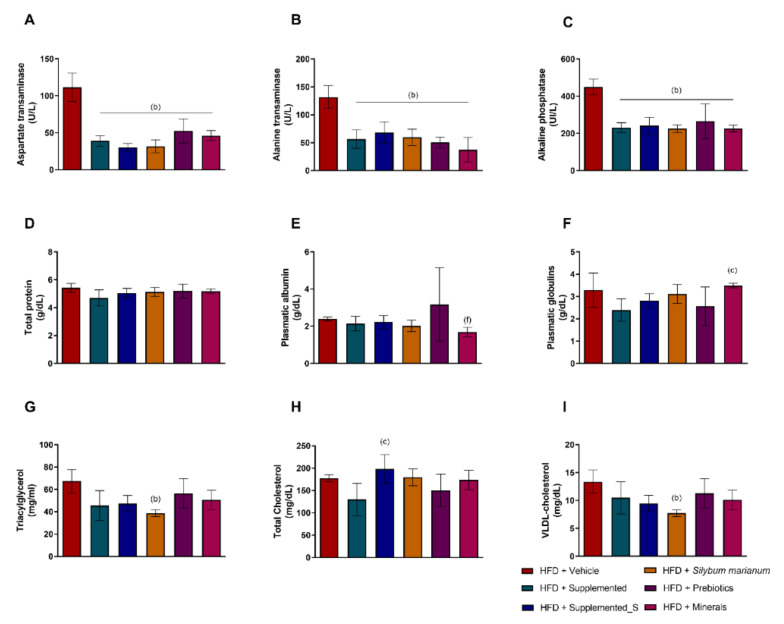
Plasmatic levels of (**A**) ALT: aspartate transaminase; (**B**) AST: alanine transaminase; (**C**) ALP: alkaline phosphatase; (**D**) total plasmatic protein; (**E**) plasmatic albumin; (**F**) plasmatic globulins; (**G**) TAG: triacylglycerol; (**H**) total cholesterol, and (**I**) VLDL-cholesterol. Significant *p* < 0.05 in one-way ANOVA followed by Tukey’s post-hoc test (n = 3–8 per group): (b) versus HFD + Vehicle; (c) versus HFD + Supplemented; (f) versus HFD + Prebiotics. HFD = high-fat diet. Values are means ± SD.

When evaluating the plasmatic biomarkers at the end of the treatment, it is possible to infer that all different supplementations were able to promote a hepatic protective effect against the HFD damage. ALT (aspartate transaminase) was significantly increased in the HFD + Vehicle group with *p* < 0.0001 compared to all other experimental groups, even though they were under the same dietary pattern (Figure 3A); likewise, AST (alanine transaminase) had the same pattern of protective effect in HFD groups (*p* < 0.0001 compared to all other experimental groups), as displayed in Figure 3B. Similarly, ALP (alkaline phosphatase) also highlights the ability of all different supplements to bring its levels down, exerting a strong positive effect compared to HFD + Vehicle (*p* < 0.0001 compared to all other experimental groups), while showing liver integrity preservation (Figure 3C). Regarding total plasmatic protein levels, no differences were observed (Figure 3D). Plasmatic albumin was reduced in the HFD + Minerals compared to the HFD + Prebiotics group (*p* = 0.0206), as shown in Figure 3E. On the other hand, plasmatic globulins levels were increased in HFD + Minerals compared to the HFD + Supplemented group (*p* = 0.0290), which is indicative of anti-inflammatory effects of the new nutraceutical (Figure 3F).

Analyzing the plasmatic lipids fraction, the decrease of TAG (triacylglycerol) and VLDL-cholesterol in the HFD + *Silybum marianum* group compared to the HFD + Vehicle group (*p* = 0.0038 and *p* = 0,0054 as shown in Figure 3G and 3I, respectively) is noteworthy. Another remarkable change is related to the plasmatic total cholesterol, which was widely modulated by the new nutraceutical. The HFD + Supplemented group displayed lower cholesterol levels even compared to HFD + Supplemented_S (*p* = 0.0145), as shown in Figure 3H. Here, we can suggest the symbiosis of nutrients promoted a stronger beneficial effect.

### 3.2. New Nutraceutical Improves Insulin Sensitivity and Liver Steatosis

**Figure 4:**
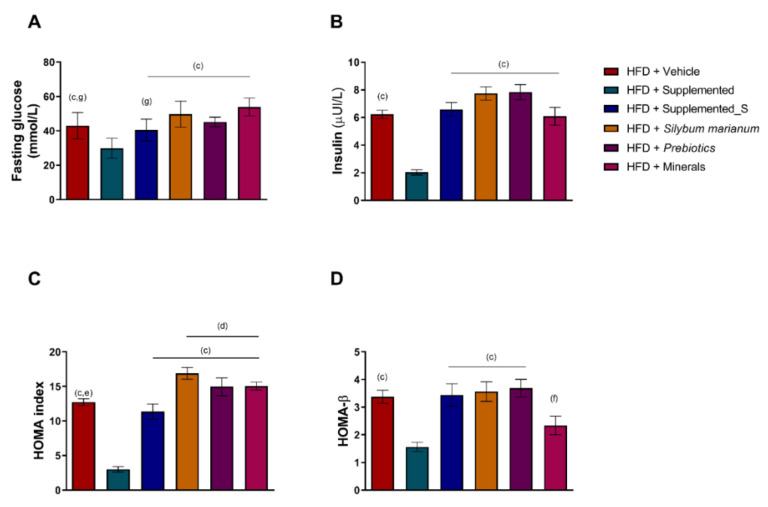
Insulin sensitivity tests. (**A**) Fasting glucose, (**B**) fasting insulin, (**C**) HOMA index, and (**D**) HOMA-β. Significant *p* < 0.05 in one-way ANOVA followed by Tukey’s post-hoc test (n = 4–8 per group): (b) versus HFD + Vehicle; (c) versus HFD + Supplemented; (d) versus HFD + Supplemented_S; (e) versus HFD + *Silybum marianum*; (f) versus HFD + Prebiotics, and (g) versus HFD + Minerals. HFD = high-fat diet. Values are means ± SD.

Fasting insulin and glycemia are both key molecules for insulin resistance and obesity-related chronic non-communicable diseases. When evaluating the fasting glucose blood levels, it is possible to conclude that HFD + Supplemented showed lower glycemic levels compared to all HFD groups (*p* = 0.0013 versus HFD + Vehicle; *p* = 0.0144 versus HFD + Supplemented_S; *p* < 0.0001 versus HFD + *Silybum marianum*; *p* = 0.0001 versus HFD + Prebiotics, and *p* < 0.0001 versus HFD + Minerals). HFD + Minerals also displayed the highest significant levels of fasting glucose compared to the HFD + Vehicle (*p* = 0.0114) and HFD + Supplemented_S (*p* = 0.0010) groups (Figure 4A). Likewise, when reviewing the insulin data, HFD + Supplemented had the lowest levels of bloodstream insulin, even under HFD exposure (*p* < 0.0001 compared to all other experimental groups), as shown in Figure 4B. In the same way, the HOMA-IR (homeostatic model assessment of insulin resistance) was significantly decreased by the new nutraceutical (HFD + Supplemented) compared to the other experimental groups (*p* < 0.0001 compared to all other experimental groups), demonstrating an effect of the insulin sensitizer. HFD + Supplemented_S also had a lower HOMA-IR compared to the isolated compounds (*p* = 0.0005 versus HFD + *Silybum marianum*, *p* = 0.0481 versus HFD + Prebiotics, and *p* < 0.0382 versus HFD + Minerals) (Figure 4C). When using the previous data to get the HOMA-β (homeostasis model assessment of β-cell function), it is possible to suggest an improvement of β-cells in the supplemented groups (HFD + Supplemented) compared to all other experimental groups, except for HFD + Minerals, which did not differ for any other group (*p* = 0.0025 versus HFD + Vehicle; *p* = 0.0016 versus HFD + Supplemented_S; *p* < 0.0007 versus HFD + *Silybum marianum,* and *p* = 0.0003 versus HFD + Prebiotics). Lastly, HFD + Minerals presented lower HOMA-β compared to the HFD + Prebiotics group (*p* = 0.0429), as shown in Figure 4D.

**Figure 5:**
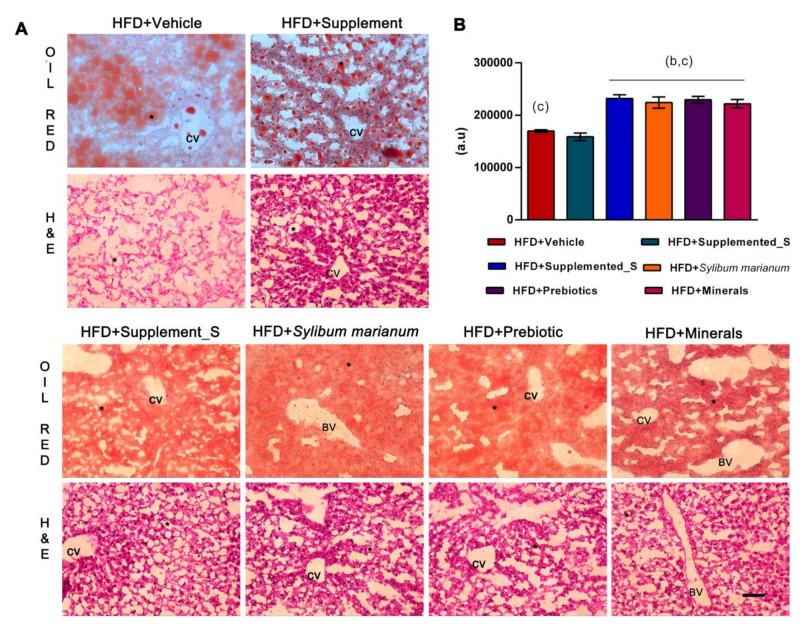
Visualization and quantification of ectopic fatty acids’ accumulation in liver samples. (**A**) Histological analysis of neutral lipids by oil red analysis and H&E staining in liver sections from mice on HFD. Each image contains a representative histological liver section image from each group. Scale bars, 50 μm; magnification is ×20; BV: blood vessel; CV: central vein; (*): lipid droplets. (**B**) RGB measurement of oil red lipid staining in liver sections. *p* < 0.05 compared with HFD + Vehicle (b) and HFD + Supplemented (c) mice versus HFD + Supplemented_S, versus HFD + *Silybum marianum*, versus HFD + Prebiotics, and versus HFD + Minerals. HFD = high-fat diet. Values are means ± SD. Significant *p* < 0.05 in unpaired *t*-test: n = 3–5 per group.

Hepatic lipid accumulation was observed in non-alcoholic fatty liver disease (NFALD) and metabolic syndrome. Beyond this, HFD + Minerals was closely related to insulin resistance [47]. We, therefore, investigated the hepatic accumulation of lipids by oil red and histological morphology by H&E (hematoxylin and eosin) staining of frozen liver sections (Figure 5A). As expected, massive amounts of lipid droplets were found in liver sections of HFD + Vehicle after 14 weeks with HFD feed, including in the central vein (CV), indicative of hepatosteatosis. The H&E stain showed loss of the liver parenchyma, besides the shape (polyhedral), and a lower hepatocytes content as observed for the weak acidophil aspect. After four weeks of oral supplementation, HFD + Supplemented demonstrated reduced lipid drops, as confirmed by the quantitative oil red analysis (Figure 5B), and the hepatocytes recovered their shape and acidophil characteristic. The control group that received the supplement showed the same phenotype (see Supplementary Figure S1).

Of note, the HFD mice supplemented with HFD + Supplemented_S and the isolated compounds (*Silybum marianum*, Prebiotics, and Minerals) did not show an improvement to their lipid content. Differing to this, the fatty acid content seems spread (as confirmed by the intense cherry color) and increased lipid drops were noted (Figure 5A,B). However, the liver parenchyma recovered after all supplements (Figure 5A).

### 3.3. Antioxidant Enzymes’ Activity Is Modulated by New Nutraceutical

**Figure 6:**
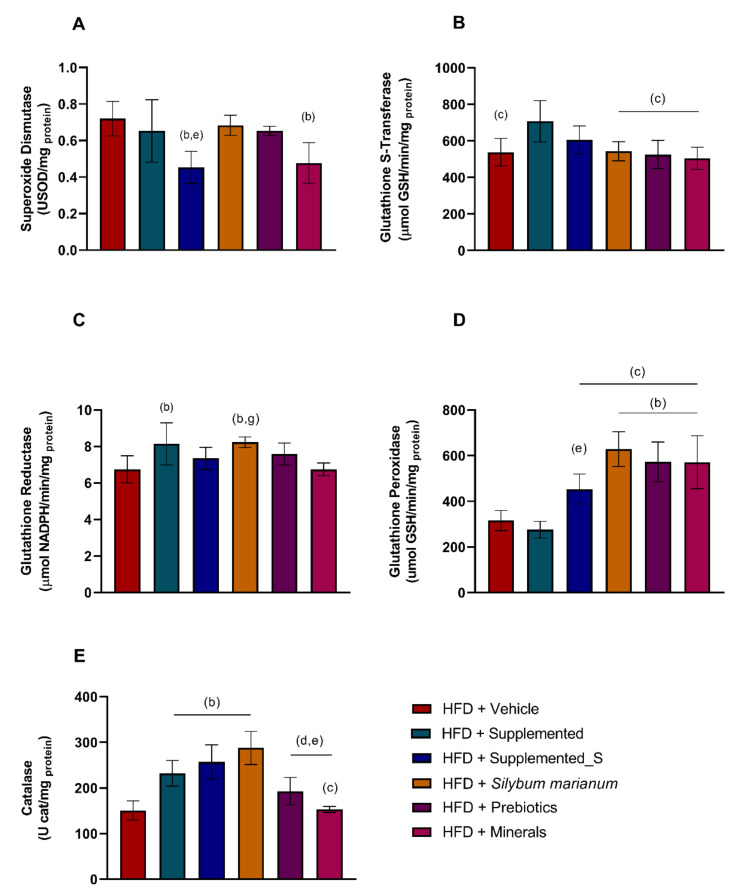
Antioxidant enzymes’ activity in liver samples. (**A**) SOD: superoxide dismutase; (**B**) GST: glutathione S-transferase; (**C**) GR: glutathione reductase; (**D**) GPX: glutathione peroxidase; (**E**) CAT: catalase. Significant *p* < 0.05 in one-way ANOVA followed by Tukey’s post-hoc test (n = 4–8 per group): (b) versus HFD + Vehicle (c) versus HFD + Supplemented; (d) versus HFD + Supplemented_S (e) versus HFD + *Silybum marianum*; and (g) versus HFD + Minerals. HFD = high-fat diet. Values are means ± SD.

Regarding the oxidative stress biomarkers, the antioxidant effect promoted by the new nutraceutical is notable. The SOD (superoxide dismutase) activity was downregulated in HFD + Supplemented_S compared to HFD + Vehicle (*p* = 0.0050) and HFD + *Silybum marianum* (*p* = 0.0240). SOD was also reduced in HFD + Minerals versus HFD + Vehicle (*p* = 0.0121), as shown in Figure 6A. The GST (glutathione S-transferase) had a large effect on HFD + Supplemented compared to HFD + Vehicle (*p* = 0.0210) and the isolated compounds (*p* = 0.0145 versus HFD + *Silybum marianum, p =* 0.0114 versus HFD + Prebiotics, and *p* = 0.0071 versus HFD + Minerals), demonstrating the augmentation of antioxidant enzyme activities (Figure 6B). GR (glutathione reductase) followed the same pattern with increased activity in the HFD + Supplemented group compared to HFD + Vehicle (*p* = 0.0398) (from CTL group, see Supplementary Figure S2). The GR activity was also higher in the HFD + *Silybum marianum* group compared to HFD + Vehicle (*p* = 0.0185) and HFD + Minerals (*p* = 0.0302), as shown in Figure 6C. The GPX (glutathione peroxidase) activity was increased in the isolated compounds (*p* < 0.0001 versus HFD + *Silybum marianum*, HFD + Prebiotics, and HFD + Minerals) compared to HFD + Vehicle. The GPX was also reduced in HFD + Supplemented compared to the other HFD groups receiving supplementation (*p* = 0.0112 versus HFD + Supplemented_S; *p* < 0.0001 compared to HFD + *Silybum marianum,* HFD + Prebiotics, and HFD + Minerals groups). Lastly, the HFD + *Silybum marianum* group had higher levels of GPX compared to the HFD + Supplemented_S group (*p* = 0.0176) (Figure 6D). Finally, the CAT (catalase) activity was increased in HFD + Supplemented (*p* = 0.0046), HFD + Supplemented_S (*p* = 0.0002), and HFD + *Silybum marianum* (*p* < 0.0001) compared to the HFD + Vehicle group. Moreover, HFD + Supplemented CAT was increased in comparison to the HFD + Minerals group (*p* = 0.0100). The CAT activity in the HFD + Supplemented_S and HFD+ *Silybum marianum* groups was also increased compared to HFD + Prebiotics (*p* = 0.0337 and *p* = 0.0003, respectively) and HFD + Minerals (*p* = 0.0006 and *p* < 0.0001, respectively) (Figure 6E). These data provide a basis to infer the antioxidant effect, as demonstrated in the supplemented groups.

### 3.4. New Nutraceutical Modulated Metabolic Homeostasis Genes in Hepatic Steatosis

**Figure 7:**
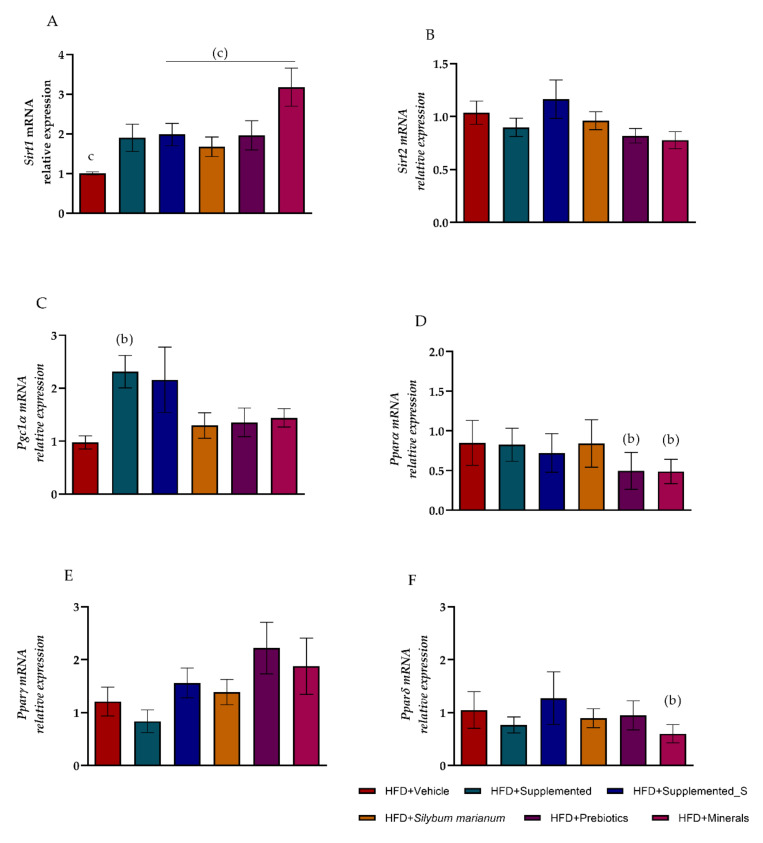
Liver mRNA expression is determined by quantitative PCR. Differences were seen in: (**A**) *Sirt1*; (**B**) *Sirt2*; (**C**) *Pgc1 alpha*; (**D**) *Pparα*; (**E**) *Pparγ*; (**F**)*Pparδ*. Significant *p* < 0.05 in one-way ANOVA followed by Tukey’s (parametric) (**A**–**C**,**E**,**F**) or Müller Dunn’s (nonparametric) (**D**) post-hoc test (n = 4–8 per group). Means and standard deviations were used to generate the effect size estimates (Hedge’s g): (b) versus HFD + Vehicle; (c) versus HFD + Supplemented. HFD = high-fat diet. Values are means ± SD. The housekeeping gene was Gapdh. Values are means ± SD.

Considering the previously observed glycemic and insulinemic profile improvement after the supplementation period, we analyzed the gene expression of *Sirt1, Sirt2, Pgc1a,* and *Ppar*s since they play an important role in regulating insulin sensitivity and adipogenesis. The *Sirt1* expression was decreased in HFD + Vehicle and HFD+*Silybum marianum* compared to HFD + Supplemented. On the other hand, the HFD + Supplemented_S, HFD + Prebiotics, and HFD + Minerals groups upregulated *Sirt1* expression, showing large effects (g.0.8) and statistical significance (*p* < 0.5) in comparison to HFD + Supplemented (Figure 7A). There was no change in *Sirt2* gene expression (Figure 7B) with Hedges *g* < 0.8, which makes the lack of supplementation effect on this specific gene clear (Hedges *g.* −0.47; 95% CI, −1.44–0.50; *p* = 0.775).

When reviewing the *Pgc1a* expression, the increase in the HFD + Supplemented group compared to HFD + Vehicle (Hedges g. 1.81; 95% CI, 0.62–3.01; *p* = 0.016) is noteworthy, suggesting a recovery following HFD metabolic damage (Figure 7C). Moreover, despite no difference in *Pparα* gene expression between the HFD + Vehicle and the HFD + Supplemented groups (Hedges g. −0.09; 95% CI, −1.07–0.89; *p* = 0.85), the *Ppar*α expression was significantly reduced by HFD + Prebiotic (Hedges g. −1.29; 95% CI, −2.35–(−0.23); *p* = 0.020) and HFD + Minerals (Hedges g. −1.44; 95% CI, −2.59–(−0.28); *p* = 0.018) compared to HFD + Vehicle (Figure 7D). Moreover, there were no differences in *Pparγ* gene expression modulation among the experimental groups (Figure 7E). The *Pparδ* gene expression was significantly reduced in HFD + Minerals compared to the HFD + Vehicle group (Hedges g. −1.46; 95% CI, −2.59–(−0.33); *p* = 0.014). Nevertheless, the HFD + Supplemented group demonstrated a large effect size on *Pparδ* expression compared to the HFD + Vehicle groups (Hedges g. −0.97; 95% CI, −1.98–0.05; *p* = 0.068), supporting the supplementation’s effectiveness (Figure 7F).

### 3.5. Symbiotic Effect of New Nutraceutical Increased Inflammatory and Anti-Inflammatory Cytokines

**Figure 8:**
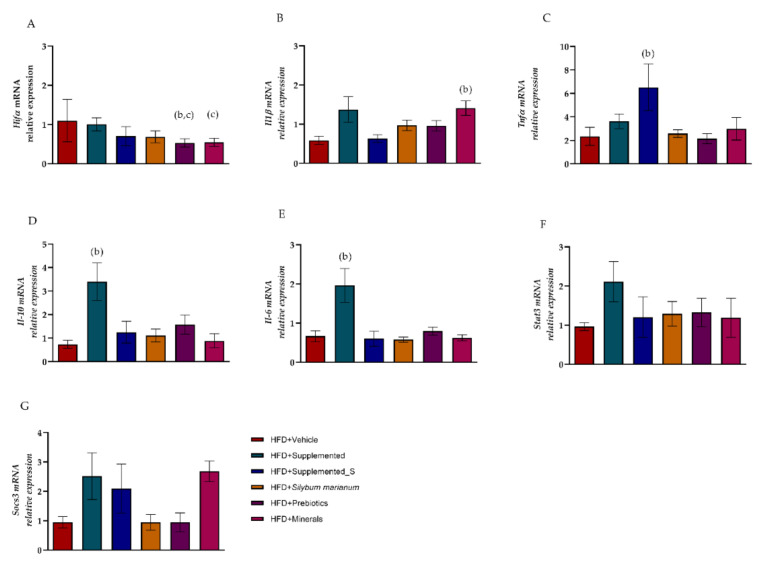
Liver expression of genes related to cytokines. Liver mRNA expression is determined by quantitative PCR. Differences were seen in: (**A**) *Hifα*; (**B**) *Il1β*; (**C**) *Tnfα*; (**D**) *Il10*; (**E**) *Il6*; (**F**) *Stat3*; (**G**) *Socs3*. Significant *p* < 0.05 in one-way ANOVA followed by Tukey’s (parametric) (**A**–**D**,**F**,**G**) or Müller Dunn’s (nonparametric) (**E**) post-hoc test (n = 4–8 per group). Means and standard deviations were used to generate the effect size estimates (Hedge’s g): (b) versus HFD + Vehicle; (c) versus HFD + Supplemented. The housekeeping gene was *B2m*. Values are means ± SD.

Another key point investigated in this study was the gene expression of inflammatory signaling pathway molecules, such as cytokines, which are directly linked to insulin resistance as well as to meta-inflammation in metabolic disorders. The *Hif1a* gene expression was significantly decreased in HFD + Prebiotics (Hedges g. −1.37; 95% CI, −2.42–(−0.33); *p* = 0.001) and HFD + Minerals (Hedges g. −1.23; 95% CI, −2.32–(−0.14); *p* = 0.031) compared to HFD + Vehicle. The HFD + *Silybum marianum* (Hedges *g*. −0.98; 95% CI, −1.97–0.00; *p* = 0.056) and HFD + Supplemented_S (Hedges g. −0.80; 95% CI, −1.88–0.28; *p* = 0.159) groups also showed a large effect on the *Hif1a* gene compared to HFD + Vehicle (Figure 8A). The gene expression of *Il1b* was increased only in HFD + Minerals compared to HFD + Vehicle (Hedges g. 2.05; 95% CI, 0.72–3.37; *p* = 0.003). Despite the lack of statistical differences, the groups receiving any sort of supplementation demonstrated a large effect on *Il1b* gene expression when compared to the HFD + Vehicle group (versus HFD + Supplemented Hedges g. 1.11; 95% CI, 0.01–2.21; *p* = 0.055; versus HFD + *Silybum marianum* Hedges *g.* 1.07; 95% CI, 0.01–2.14; *p* = 0.055; versus HFD + Prebiotics Hedges *g.* 1.01; 95% CI, −0.05–2.07; *p* = 0.068), as shown in Figure 8B. The *Tnfa* gene expression was significantly increased with a large effect in the HFD + Supplemented_S group compared to HFD + Vehicle (Hedges g. 1.22; 95% CI, 0.08–2.36; *p* = 0.042). However, the HFD + Supplemented group also displayed a large effect on *Tnfa* expression compared to the HFD + Vehicle group (Hedges g. 0.62; 95% CI, −0.36–1.60; *p* = 0.228), as shown in Figure 8C.

On the other hand, the anti-inflammatory cytokine *Il-10* showed a sharp increase in the HFD + Supplemented group in comparison to HFD + Vehicle (Hedges g. 1.49; 95% CI, 0.40–2.58; *p* = 0.001). In the same way, *Il-6* gene expression demonstrated similar behavior, with a substantial effect on the HFD + Supplemented group when compared to HFD + Vehicle (Hedges g. 1.43; 95% CI, 0.31–2.54; *p* = 0.031), as shown in Figure 8D,E (from CTL group, see Supplementary Table S3).

Although *Stat3* and *Socs3* did not demonstrate statistical significance between the supplement or the isolated compounds, the HFD + Supplemented group showed a noteworthy difference compared with HFD + Vehicle when we considered the higher effect size than Hedges g. 0.8 (Hedges g. 1.22; 95% CI, 0.10–2.34; *p* = 0.599 and Hedges g. 0.96; 95% CI, −0.09–2.00; *p* = 0.996, respectively). Likewise, HFD + Supplemented_S and HFD + Minerals modulated *Socs3* gene expression in effect size analysis (Hedges g. 1.00; 95% CI, −0.20–2.20; *p* > 0.999 and Hedges g. 2.53; 95% CI, 1.06–3.99; *p* = 0.134), as demonstrated in Figure 8F,G.

## 4. Discussion

Currently, using natural products or their derivatives for the prevention or treatment of assorted diseases represents a reasonable way to use natural resources and branded technology as self-sufficiently viable alternatives. The development of new products containing different elements and natural derivatives, aiming to create a symbiotic effect among them acting holistically on the human body, is a challenge to be overcome. Our recently published findings show the effects of a new supplement, with nutraceutical properties, in a diet-induced obesity mouse model [5]. Our results have shown a symbiotic effect between β-glucan yeast, prebiotic, minerals (selenium, zinc, magnesium), and *Silybum marianum* (L.) Gaertn. (silymarin) on factors involved in mitochondrial biogenesis (*Pgc-1α*, TFAM, SIRT), inflammation (NFκB), and antioxidant enzymes (SOD), which recovered glycemic and lipid homeostasis and reduced insulin resistance and hepatic steatosis in an obese mouse model. Although the components of the new nutraceutical have already had their effects proven for restoring several parameters when evaluated in isolation, they are often administered in high doses or drug concentrations [31,32,33,34]. Thus, in the presented study, we evaluated the expression of genes involved in the effects observed by our group in the improvement of hyperglycemia, insulin resistance, inflammation, and hepatic steatosis that only occurred when using the new nutraceutical, or if the use of a variation of the supplement without *Silybum marianum* (silymarin) and its components grouped by product class could also modulate the parameters herein investigated.

Type-2 diabetes (T2D) is characterized by chronic hyperglycemia, resulting from the breakdown of metabolic homeostasis, involving mainly glucose and fatty acids’ metabolism [53]. The typical hyperglycemia triggered by insulin resistance is closely related to obesity and NAFLD development [35,54]. The increased inflammatory cytokine expression by the white adipose tissue alongside ROS production leads to low-grade chronic inflammation, also called meta-inflammation. The systemic release of inflammatory cytokines such as *IL-6* and TNF-α directly contributes to insulin resistance. It is well-known that the increase of insulin and glucose in the bloodstream triggers systemic inflammation and oxidative stress, contributing to non-communicable chronic diseases’ impairment, such as T2D and obesity [21,22,54]. The new nutraceutical constitution test here comprises the key cofactors minerals—magnesium, zinc, and selenium—for carbohydrate metabolism and modulation of oxidative stress. Magnesium participates in the glycolytic pathway and the regulation of mitochondrial function [55]. Moreover, zinc acts on the insulin pathway [56], and selenium participates in structuring antioxidant enzymes such as GPX (glutathione peroxidase) and selenoprotein P, which are involved in the insulin signaling pathway [57]. Furthermore, the *Silybum marianum*, besides its well-recognized hepatoprotective effects, also increases the insulin receptor (IR) sensitivity [58]. Though yeast β-glucan is an immunomodulatory molecule, Cao et al. demonstrated its hypoglycemic effect by suppressing sodium-glucose transporter-1 expression in obese/type-2 diabetes mice [59]. Additionally, fructo-oligosaccharides (FOS) [60], galacto-oligosaccharides (GOS) [61], and mannan oligosaccharides (MOS) [62] are insoluble prebiotic fibers capable of modulating glucose, along with lipids levels like cholesterol and triacylglycerols, by decreasing their absorption at the intestinal level. As well as their byproducts, which have a systemic effect, decreasing lipogenesis signaling mainly affects the hepatic level. This happens due to the short-chain fatty acids (SCFA), namely propionate and butyrate, produced by the gut microbiota fermentation of soluble fibers. This SCFA can promote improvement in metabolism, body weight, energy homeostasis, and glycemic response through intestinal gluconeogenesis [63]. Although a recent meta-analysis has shown that no fiber has stood out in terms of its effects on glycemic metabolism, it is generally agreed that a daily intake of 15 to 35g of fiber improves or reduces diabetes mortality [64]. On the other hand, large amounts of FOS and GOS intake can cause the opposite effects, with increased blood glucose and microbiota reshaping [65].

Weight loss associated with caloric restriction generally leads to glycemic control, decreased visceral fat, and improved insulin resistance [66,67]. All supplemented groups showed a reduction in body weight gain, which demonstrates an increase in satiety across all supplements. However, only the new nutraceutical was able to reduce the BMI. Though Supplemented_S led to a lower intake compared to the others, this was not an advantage for the body measure parameters evaluated.

To assess whether the new nutraceutical, Supplemented_S, and isolated compounds *Silybum marianum* (silymarin), prebiotics (yeast-β glucan, FOS, GOS, and MOS), and minerals (zinc, magnesium, and selenium) were able to cause liver damage, we evaluated the main liver enzymes, such as ALT, AST and alkaline phosphatase [68]. Considering that albumin is a predictive factor of NAFLD worsening [69], we also proceeded with a liver protein profile—total protein, albumin, and globulin—to check if the supplements would have any effect, and even whether in our mouse model of hepatic steatosis could be altered. Yet, no changes were noteworthy. Only circulating lipoproteins TAG and VLDL were decreased in the obese group supplemented with *Silybum marianum,* as expected when considering the known hepatoprotective effect of silymarins.

Hyperglycemia, insulinemia, and insulin resistance are inherent in T2D, which in turn, is associated with obesity [53]. The insulin resistance (IR) can be assessed by homeostasis model assessment (HOMA-index), as well as the functional capacity of beta cells (β-cell function—HOMA-β), which are measures used as the standard for clinical assessment of IR. Though it presents controversies [70], pancreatic β-cells’ malfunction is part of the pathophysiology of type-2 diabetes [71]. Our group demonstrated that the new nutraceutical was able to reduce postprandial and fasting glycemia and insulin levels, which was reflected in enhanced inflammation, hepatic steatosis, and redox homeostasis recovery [5]. As the new composition contains elements that by themselves can recover carbohydrate metabolism and insulin sensitivity [31,32,33,34], we evaluated if a new variation of the new nutraceutical without silymarin (Supplemented_S) or the isolated compound groups would be able to improve the glycemic homeostasis and insulin resistance. Although the improvement in the HOMA index and β-cell function is associated with weight loss in humans [71]—a condition that was observed in all supplemented groups—unexpectedly, only the new nutraceutical was able to recover the glycemia and insulin sensitivity, despite integrating the homeostasis assessment with the HOMA index and HOMA-β. This was surprising since the concentrations of the elements selected in the supplements were below those recommended for daily intake. Thus, this result corroborates our hypothesis that the symbiotic effect between the natural and derivative compounds of the supplement products can improve metabolic and redox homeostasis.

Oxidative stress is a result of imbalance between the antioxidant systems and the generation of reactive species (ROS) [72]. Type-2 diabetes (hyperglycemia) and obesity (free fat acids—FFA) are closely associated with increased mitochondrial ROS generation [73], which in turn, decreases the insulin content and glucose-stimulated insulin secretion of β-cells [74]. It is already known that in T2D and hepatic steatosis, there is a decrease in the activity of the antioxidant enzymes SOD, catalase, GPX, glutathione reductase (GR), and hepatic glutathione S-transferases (GSTs), as well as minerals like magnesium, zinc, iron, and selenium [72,74,75,76], although there is not a consensus [77]. The new nutraceutical has already demonstrated its antioxidant effect by increasing SOD expression in the HFD group, as well as reducing oxidative stress secondary metabolites (MDA and OxyBlot) [5]. The glutathione transferases are ubiquitous enzymes distributed across cells’ cytoplasm, microsomes, and mitochondria, with an important role in the phase-2 biotransformation of xenobiotics, besides participating in the reduction of glutathione (L-ɣ-glutamyl-L-cysteinyl glycine, GSH), along with glutathione reductase [78], and in the detox of lipid peroxidation byproducts, such as 4-hydroxynonenal [79]. Dastidar et al. demonstrated that liver glutathione S-transferase was decreased in a diet-induced obesity model, which resulted in glucose intolerance, suggesting that the enzyme may be a pathway to be considered in the prevention or treatment of diabetes [80]. Recovering redox homeostasis by reducing the generation of ROS through activating antioxidant enzymes has been proved to be an accessible way to recover insulin sensitivity [81] and to reduce fat accumulation in the liver [80,82]. The new nutraceutical was the only one to increase GST, GR, and catalase activity in the supplemented groups compared to the HFD-Vehicle group. The GR and catalase were also modulated by *Silybum marianum* (silymarin), in agreement with previous reports [82]. However which factors would be working together with antioxidant enzymes to improve redox and glycemic homeostasis?

In this sense, *Pgc-1α* (PPARγ coactivator 1) is associated with decreased production and release of triacylglycerols in the liver [83], acting as a key regulator of mitochondrial biogenesis, reactive oxygen species’ (ROS’) detoxification, energy, and oxidative metabolism, besides integrating the circadian clock as a key component [84,85]. *Pgc-1α* and SIRT 1 (NAD-dependent deacetylase sirtuin1) are also involved in the expression of the antioxidant enzyme catalase [76]. Koo and collaborators described how *Pgc-1α* promotes insulin resistance through PPAR-α-dependent induction of TRB-3 during fasting periods. Induced PGC-1 deficiency in mice demonstrated threefold higher triglyceride levels and hypoglycemia. This is probably by fatty acid oxidation enzymes and gluconeogenesis decrease [86]. *Pgc-1α* was also downregulated in obesity and NAFLD [87], and as a consequence, we observed decreasing β-oxidation, oxidative phosphorylation, mitochondrial biogenesis, and insulin resistance [85,87]. Although there is some controversy regarding the role of *Pgc-1α* in energy homeostasis [88], the new nutraceutical was the only one among the supplementations applied to increase the expression of the *Pgc-1α* gene, corroborating its protein expression in a previous work published by our group [5].

The family of nuclear peroxisome proliferator-activated receptors (PPARs) is subordinate to *Pgc-1α*. Thus, we verified the supplements’ modulation of the *Pparγ, Pparα,* and *Pparδ* genes that actively participate as sensors and regulators of lipid metabolism since they act as therapeutic targets for hypoglycemic agents, such as rosiglitazone, and lipid-lowering agents such as fenofibrate, improving hyperglycemia, insulin resistance, and liver fat accumulation [89]. The improvement observed in insulin resistance and hepatic fat accumulation does not seem to be due to the gene modulation of *Ppars* via *Pgc-1α* by the new nutraceutical.

Insulin signaling is impaired by metainflammation due to the increase in body fat mass [90]; however, adipose tissue seems to wield under insulin resistance with a pleiotropic effect [91,92] by the production of bioactive molecules that behave like hormones, known as adipokines [90]. The production of adiponectin in adipose tissue, and AdipoR2′s high level of expression in the liver, increase insulin sensitivity via activation of AMPK and PPAR-α [92,93]. Unlike this, the adipokines such as leptin, TNFα, interleukin 1β (IL-1β), and interleukin 6 (*IL-6*) impaired the insulin sensitivity commonly with an antagonist effect under adiponectin [91]. *IL-6* is a cytokine/adipokine involved in inflammatory signaling and glucose and lipid pathways, in addition to being activated by the STAT3 transcription factor (signal transducer and activator of transcription 3) and suppressed by SOCS3 (suppressor of cytokine signaling 3) [94,95], which is associated with insulin resistance [96] by inhibiting IRS-1 phosphorylation in adipose tissue [96,97]. In contrast, *IL-6* derived from hepatic macrophages was able to activate IRS-2, improve insulin sensitivity, and was upregulated by adiponectin gene expression [98]. Interestingly, *Pgc-1α* was also able to improve glycemic homeostasis via IRS-2 [99]. Nevertheless, Matthews et al. demonstrated that *IL-6*-deficient mice fed with HFD showed no improvement in insulin resistance and also gained weight and had liver fat accumulation [100], although controversial studies must be noted [22,101].

Interleukin 10 (*IL-10*) is the most important anti-inflammatory cytokine and plays an important role in maintaining the balance of the immune system under inflammatory or infectious conditions [102]. A low level or hyporesponsiveness of this cytokine is associated with type-2 diabetes [103] and obesity [104]. *IL-10* is a protective factor for type-2 DM via STAT3 activation [21]. Gao et al. showed that overexpression of *IL-10*, in an HFD model, improved insulin sensitivity and prevented glucose intolerance, in addition to reducing the accumulation of ectopic fat and decreasing the expression of inflammatory cytokine genes [105]. *Pgc-1α* was also able to modulate *IL-10* expression, improving insulin resistance and hepatic steatosis [21].

It is common sense that *IL-6* has a proinflammatory function [95] and *IL-10* acts as an anti-inflammatory molecule [102], although both have a pleiotropic effect. These same cytokines seem to act symbiotically in controlling inflammation and in regulating the signaling of hormones such as insulin and leptin in the central nervous system [106], muscles [107], and vascular system [108]. In our study, we described for the first time this symbiosis between the genes of *Il-6*, *Il-10*, and *Pgc-1α* o in the liver of Control (Table S3) and HFD animals supplemented with a composition of different classes of natural derivatives containing minerals, silymarin, and prebiotics. The result of a symbiosis between cytokines and the transcription factor increased insulin sensitivity, reduced blood glucose and insulin, and improved liver steatosis and antioxidant activity. With complete supplementation of natural derivatives, the new nutraceutical was able to recover metabolic homeostasis and redox in a T2D obesity model. We believe that these changes start in the intestinal environment, with the modulation of the microbiota and improvement in the absorption of micro and macronutrients.

We have confirmed in the present study the effects of supplementation with the composition in its entirety, and we suggest that this improvement is mainly due to the gene and protein modulation of a factor involved in the regulation of metabolism, *Pgc-1α*. Yet, we are aware of the limitations of the study as we only evaluated the gene expression of several factors. So, future investigations into the protein phenotype of the analyzed pathways are suggested.

In conclusion, we observed that although the isolated use of the formulation components had some specific effects on some of the parameters studied, only the composition containing all the components, the new nutraceutical, was able to recover homeostasis due to its symbiotic action among the components. Thus, we believe that the new nutraceutical is a new nutraceutical category with positive effects on several parameters of type-2 diabetes in obese patients.

## 5. Patents

The formulation of the supplement (patent number: BR 10 2020 016,156 3) can be found at Revista de Propriedade Industrial nº 2667, accessed on 15 February 2022.

## Figures and Tables

**Table 1:** Specific supplement composition described by experimental group.

Groups	Components
Supplemented	Zinc (Zn) 0.63%; selenium (Se) 0.003%; magnesium (Mg) 4.35%; FOS 49.69%; GOS 31.05%; yeast β-glucans (*S. cerevisiae*) 11.18%; silymarin extract 3.11%
Supplemented_S	zinc (Zn) 0.63%; selenium (Se) 0.003%; magnesium (Mg) 4.35%; FOS 49.69%; GOS 31.05%; yeast β-glucans (*S. cerevisiae*) 11.18%
*Silybum marianum* (silymarin)	silymarin extract 3.11%
Prebiotics	FOS 49.69%; GOS 31.05%; yeast β-glucans (*S. cerevisiae*) 11.18%
Minerals	Zinc (Zn) 0.63%; selenium (Se) 0.003%; magnesium (Mg) 4.35%

## Data Availability

The raw data of this research are accessible by contacting the corresponding author.

## Supplementary Materials

The following supporting information can be downloaded at: https://www.mdpi.com/article/10.3390/antiox11030447/s1, Figure S1. Visualization and quantification of neutral lipids by OIL RED analysis and H&E staining in liver sections from Control + Vehicle and Control + Supplemented mice; Figure S2. Antioxidant enzymes’ activity in liver samples of control diet groups; Table S1. Diet composition; Table S2. Mus musculus genes used in the RT-qPCR analysis, number of genes in the GenBankTM platform (http://www.ncbi.nih.gov/gene, accessed on 31 January 2022), and gene sequences of forward and reverse primers of each gene studied; Table S3. Liver expression of genes related to cytokines and metabolic homeostasis.
