# Supplementary material for: The Symbiotic Effect of a New Nutraceutical with Yeast β-Glucan, Prebiotics, Minerals, and Silybum marianum (Silymarin) for Recovering Metabolic Homeostasis via Pgc-1α, Il-6, and Il-10 Gene Expression in a Type-2 Diabetes Obesity Model
Source: Antioxidants (Basel). 2022 Feb 23;11(3):447. doi: 10.3390/antiox11030447 (PMC8944780; doi:10.3390/antiox11030447)
Supplement: Supplementary file 1 [file antioxidants-11-00447-s001.zip › antioxidants-1599027-supplementary.pdf]

Supplementary Material

Table S1. Diet composition.

	Diet (g/kg)	
**Components**	**Control (AIN93M)**	**High-fat diet (G)**
**Maize starch**	465.7	175.45
**Sucrose**	100	110
**Casein**	140	220
**Dextrinized starch**	155	110
**Soy oil**	40	44
**Lard**	-	330
**Microcrystalline cellulose**	50	55
**AIN 93 vitamin blend**	10	11
**AIN 93 mineral mix**	35	38.5
**L-cystine**	1.8	3.3
**Choline bitartrate**	2.5	2.75
**Butylhydroquinone**	0.008	0.031
	**Macronutrients**	
**Carbohydrates (%)**	79.6	39.7
**Protein (%)**	15.9	22.7
**Lipids (%)**	4.5	37.6
**Energy (Kcal/g)**	3.5	5.2

Table S2. *Mus musculus* genes used in the RT-qPCR analysis, number of genes in the GenBank™ platform (http://www.ncbi.nih.gov/gene), and gene sequences of forward and reverse primers of each gene studied.

**Gene**	**Gene no.**	**Forward**	**Reverse**
il-6	16193	TAGTCCTTCCTACCCCAATTTCC	TTGGTCCTTAGCCACTCCTTC
il-10	16153	GCTCTTACTGACTGGCATGAG	CGCAGCTCTAGGAGCATGTG
il-1β	16176	GGAGAACCAAGCAACGACA	TGGGATCCACACTCTCCA
stat3	20848	ACCCAACAGCCGCCGTAG	CAGACTGGTTGTTTCCATTCAGA
socs3	12702	CACCTGGACTCCTATGAGAAAGTG	GAGCATCATACTGATCCAGGAACT
hif1α	15251	AGTCAGCAACGTGGAAGGT	CGTCATGGGTGGTTTCTTG
tnfα	21926	TCTACTGAACTTCGGGGTGA	GATCTGAGTGTGAGGGTCTGG
sirt1	93759	GCTGACGACTTCGACGACG	TCGGTCAACAGGAGGTTGTCT
sirt2	64383	GCCTGGGTTCCCAAAAGGAG	GAGCGGAAGTCAGGGATACC

pgc1a	19017	TATGGAGTGACATAGAGTGTGCT	CCACTTCAATCCACCCAGAAAG
pparα	19013	TGCAATTCGCTTTGGAAGAA	CTTGCCCAGAGATTTGAGGT
pparγ	19016	TCGCTGATGCACTGCCTATG	GAGAGGTCCACAGAGCTGATT
pparδ	19015	AGCCACAACGCACCCTTT	ACCAGCTGTTTCCACACCA
*srebp1*c	20787	TGGACCACAGAAAGGTGGA	ATGGCCTTGTCAATGGAACT
b2m	12010	TTCTGGTGCTTGTCTCACTGA	CAGTATGTTCGGCTTCCCATTC
gapdh		TGTGTCCGTCGTGGATCTGA	TTGCTGTTGAAGTCGCAGGAG

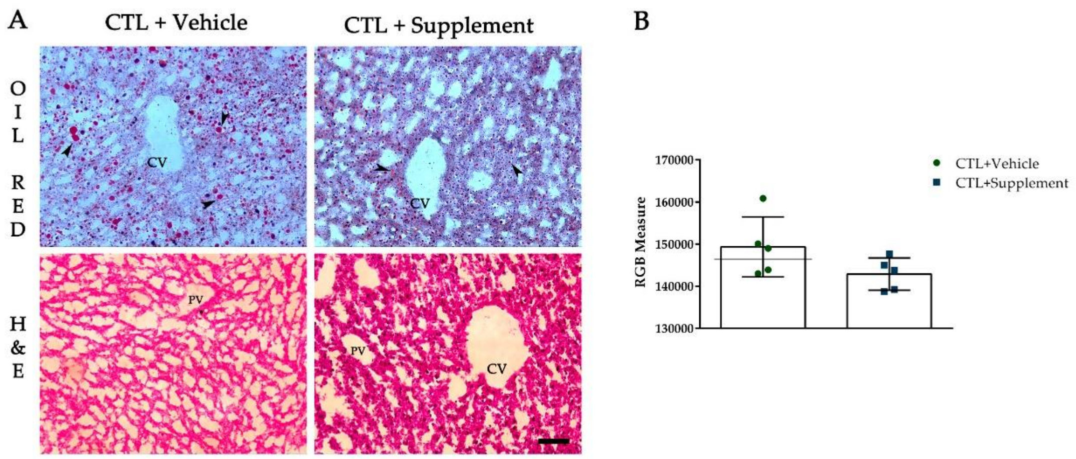

Figure S1. Visualization and quantification of neutral lipids by OIL RED analysis and H&E staining in liver sections from Control+Vehicle and Control+Supplemented mice. Scale bars, 50 µm; magnification is ×20; n = 5 per group. RGD measurement. $P < 0.05$ compared with Control+Vehicle and Control+Supplemented. Values are means ± SD. CTL=control.

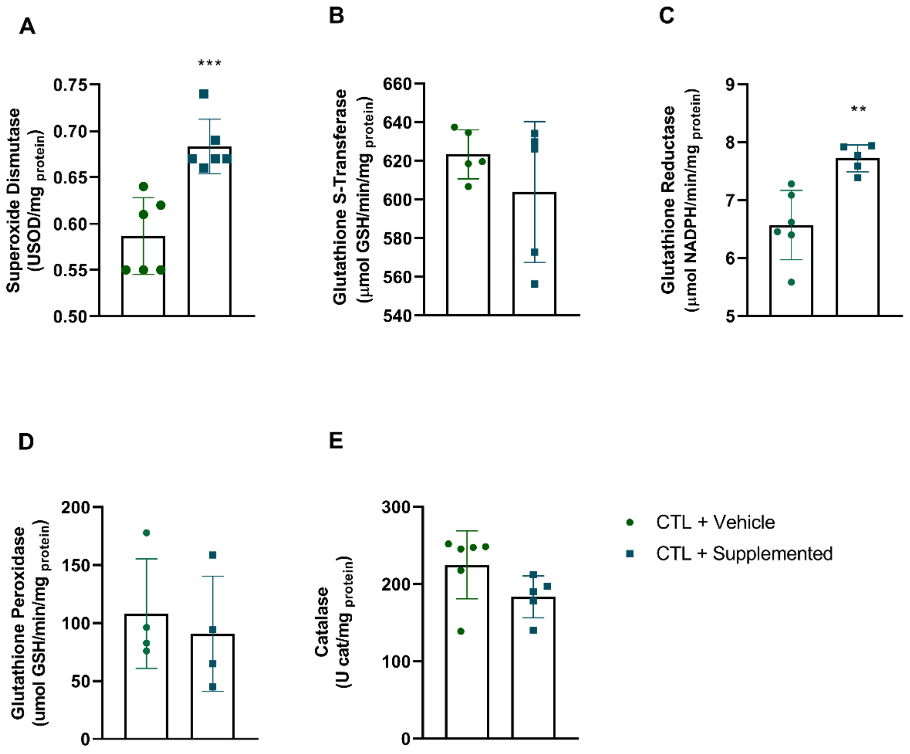

Figure S2. Antioxidant enzymes' activity in liver samples of control diet groups. (A) SOD: superoxide dismutase; (B) GST: glutathione S-transferase; (C) GR: glutathione reductase; (D) GPX: glutathione peroxidase; (E) CAT: catalase. ** Significant p < 0.01 and *** significant p < 0.001 in unpaired *t*-test (n=4–8 per group). CTL=control.

Table S3. Liver expression of genes related to cytokines and metabolic homeostasis. Liver mRNA expression is determined by quantitative PCR. Differences were seen when comparing versus CTL+Vehicle and versus CTL+Supplemented (n= 4–8 per group). The data were classified as parametric or nonparametric based on the Shapiro–Wilks test. For parametric data, comparisons between two groups were performed using the *t*-test with or without Welch's correction. Means ± standard deviation (SD) were used to generate the effect size estimates (Hedge's g). CTL=control.

	CTL vs. CTL+Supplemented
Sirt1	(Hedges *g*. 0.08; 95% CI, -0.90–1.07; *P* = 0.871)
Sirt2	(Hedges *g*. 0.11; 95% CI, -0.85–1.06; *P* = 0.829)
Pgc1a	(Hedges *g*. 0.56; 95% CI, -0.41–1.54; *P* = 0.266)
Pparα	(Hedges *g*. 0.58; 95% CI, -0.40–1.55; *P* = 0.463)
Pparγ	-
Pparδ	(Hedges *g*. 0.33; 95% CI, -0.66–1.31; *P* = 0.527)
Hif1α	(Hedges *g*. -0.72; 95% CI, -1.75–0.30; *P* = 0.178)
Il1β	**(Hedges *g*. -1.56; 95% CI, -2.76–0.36; *P* = 0.007)**
Tnfα	(Hedges *g*. -1.13; 95% CI, -2.35–0.09; *P* = 0.073)
Il10	**(Hedges *g*. 2.06; 95% CI, 0.84–3.28; *P* = 0.001)**
Il6	**(Hedges *g*. 1.84; 95% CI, 0.64–3.05; *P* = 0.025)**
Stat3	(Hedges *g*. -0.62; 95% CI, -1.63–0.38; *P* = 0.236)
Socs3	(Hedges *g*. 0.74; 95% CI, -0.28–1.76; *P* = 0.128)
